# A Survey of Body Sensor Networks

**DOI:** 10.3390/s130505406

**Published:** 2013-04-24

**Authors:** Xiaochen Lai, Quanli Liu, Xin Wei, Wei Wang, Guoqiao Zhou, Guangyi Han

**Affiliations:** 1 Faculty of Electronic Information and Electrical Engineering, Dalian University of Technology, No. 2 Linggong Road, Ganjingzi District, Dalian 116024, China; E-Mails: weixin@mail.dlut.edu.cn (X.W.); wangwei@dlut.edu.cn (W.W.); 2 School of Software, Dalian University of Technology, Economy and Technology Development Area, Dalian 116620, China; E-Mails: zhou.guo.qiao@mail.dlut.edu.cn (G.Z.); hanguangyi@mail.dlut.edu.cn (G.H.)

**Keywords:** body sensor network, sensor, data fusion, network communication, practical application of BSNs

## Abstract

The technology of sensor, pervasive computing, and intelligent information processing is widely used in Body Sensor Networks (BSNs), which are a branch of wireless sensor networks (WSNs). BSNs are playing an increasingly important role in the fields of medical treatment, social welfare and sports, and are changing the way humans use computers. Existing surveys have placed emphasis on the concept and architecture of BSNs, signal acquisition, context-aware sensing, and system technology, while this paper will focus on sensor, data fusion, and network communication. And we will introduce the research status of BSNs, the analysis of hotspots, and future development trends, the discussion of major challenges and technical problems facing currently. The typical research projects and practical application of BSNs are introduced as well. BSNs are progressing along the direction of multi-technology integration and intelligence. Although there are still many problems, the future of BSNs is fundamentally promising, profoundly changing the human-machine relationships and improving the quality of people's lives.

## 1. Introduction

As important public network applications, BSN applications are in great demand in medical care [1–3], sports and entertainment [4–6], the military-industrial sector [7], and the social public field [8–10], and BSNs have gradually become a research hotspot. BSNs are a kind of WSN which is formed by physiological parameter sensors placed in the human body, on the body surface or around the body. The main techniques it covers are sensors, data fusion, and network communication. It is not only a new type of universal health care, disease monitoring, and prevention solution, but also an important component of the so-called Internet of Things. Its main purpose is to provide an integrated ubiquitous computing hardware, software, and wireless communication technology platform, and an essential condition for the future development of ubiquitous health care monitoring systems [11].

BSNs originated from WSNs, so there are many similarities between them. However, the characteristics are correspondingly different because of their different application purposes. Firstly, considering network deployment, WSNs can be deployed to inaccessible environment, such as forests, swamps or mountains. Many redundant nodes are placed in the environments mentioned above to solve the problem of node failures, so node density is higher, whereas BSN nodes are deployed in, on or around the human body, so the total number of nodes is generally up to a few dozens. Each node ensures the accuracy of monitoring results by its robustness [12]. Secondly, considering attributes, nodes in WSNs perform the same functions, and have the same properties. The size of nodes is not very critical. Once the node is deployed, it will probably no longer need to be moved. According to the different physical signals collected, BSN applications have different sensor types [13]. Moreover, the requirements of BSN node design are relatively high. The node size must be small enough, and the nodes need to have high wearability and high biocompatibility [14]. Due to the locations the nodes are deployed, they will move as the human body moves. Thirdly, considering energy supply, WSNs and BSNs can be battery-powered. The former, deployed outdoors, can also be powered by wind energy or solar energy, while the latter can also be powered by kinetic energy and heat [15,16]. Finally, considering data transmission, the transfer rates of WSNs are almost the same, but those of BSNs are different, as the data type and channel assignment are different among nodes on the body surface and in the body [13]. Additionally, BSNs deployed in the human body are for monitoring human physiological data, which are subject to user's personal safety and privacy protection issues. Therefore, QoS and the real-time prosperity of data transmission must be considered [17,18].

**Figure 1.**
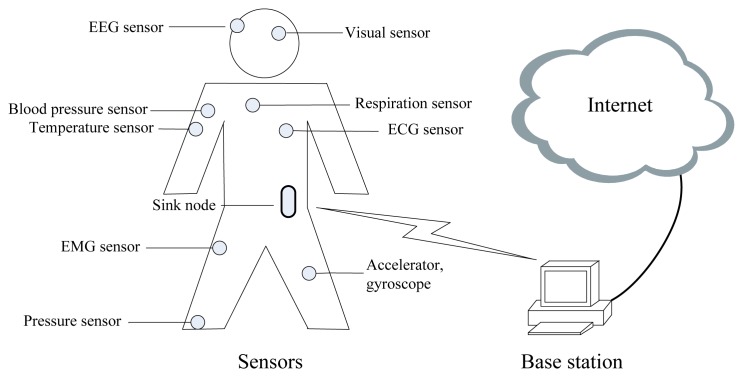
Architecture of a BSN.

The general architecture of a BSN is shown in Figure 1. Sensor nodes which are placed in the body collect physical data and perform preliminary processing. The data are gathered by a sink node and then transmitted to a base station in order to share over the Internet, which is the basis of many applications, including health care systems, social welfare, patients and immediate service, diagnosis services by doctors and medical experts, and emergency treatment systems, *etc*.

**Figure 2.**
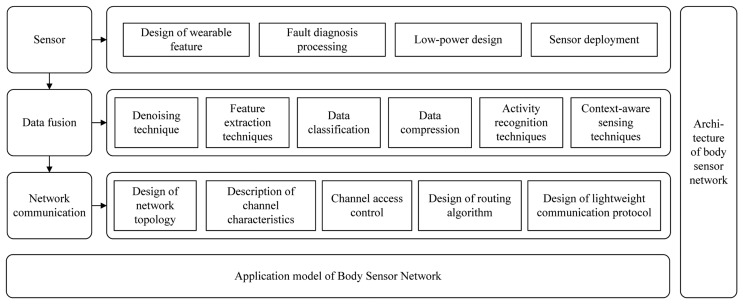
Main research areas in BSNs.

Nowadays BSN research still faces many key technical challenges. Figure 2 summarizes the main research areas of BSNs. The research work on sensor design and use mainly focuses on the wearability of sensor nodes [19], the capacity of fault diagnosis and fault treatment [20], energy consumption [21] and sensor deployment [22], *etc*. In the aspect of data fusion, research mainly includes the design and implementation of denoising [23], feature extraction [24], data classification [25], data compression [26], and other key technologies. A growing number of scholars combine situational awareness and data fusion technologies in the activity recognition area [27,28]. In the aspects of network communication, research mainly focuses on the problems of network topology design [29], channel characterization [30], channel access control [31,32], routing algorithm design [33], and lightweight communication protocols design, *etc.* These key technologies must be considered when building a complete BSN system. They are not only of great research value, but also of important practical value.

Existing surveys have made detailed investigations on BSN architecture, signal processing, communication protocols, context awareness and QoS. Reference [12] mainly focused on propagation of information, communication protocols, QoS and security. Moreover, it gave some examples of medical monitoring based on BSN. In Reference [13] the authors provided a detailed research on sensor devices, physical layer, MAC layer and radio technologies, then made a classification on body sensor projects and summarized some open research issues on multi-aspects. In Reference [34], researchers presented an overview of BSN in enabling pervasive healthcare and assistive environments, listing many applications of sensor devices and communication protocols. The authors also analyzed current obstacles and future open issues from an overall angle. Reference [35] placed emphasis on network communication, discussing the issues of physical layer, MAC layer, network layer and routing protocols in detail. Different from the above papers, we set forth our views from the perspective of system design, with a detailed explanation of the research status of three aspects: sensor, data fusion, and network communication. In each section, we summarize the issues which need to be considered in design phase, listing some typical related cases. We also present the trends of development and technical challenges. In addition, we list some key research achievements of BSNs in various fields, such as medicine, social welfare, sports, and man-machine interfaces. Moreover, we have provided a detailed analysis of trends and challenges of BSNs from a systemic perspective.

The remainder of this paper is organized as follows: Section 2 describes the various types of sensors and hotspots in BSNs. Section 3 explores the development of data fusion. Section 4 discusses BSN network topology and communication between layers. In Section 5, we introduce the typical applications in various fields and the system design problems to be considered in BSNs. Section 6 briefly concludes this paper.

## 2. Sensors

Sensors are the key components of BSN, as they connect the physical world with electronic systems. They are mainly used to collect the information about physiology and the surrounding environment. Sensor nodes, which have a sensor as their main part, are responsible for processing information by format conversion, logical computing, data storage, and transmitting. One sensor node generally comprises a sensor module, processor module, wireless communication module, and power supply module [36]. The sensor module is responsible for collecting the status of measurands and converting data from physical quantities to electrical signals. The processor module is responsible for controlling the sensor nodes. The wireless communication module, consisting of network layer, MAC layer and wireless transceiver in the physical layer, is responsible for communication among sensors and computers. The power supply module is responsible for providing energy for entire the sensor node.

### 2.1. State-of-the-Art Research on BSN Status

In recent years, with the development of BSN applications in many fields, electrocardiograph (ECG) sensors, accelerometers, pressure sensors, and respiration sensors are gradually becoming the hotspots of BSN sensor research. A large amount of research has been done on improving the wearability of sensor nodes, and optimizing data processing algorithms. An in-depth study has been made on problems such as energy control, fault diagnosis, and sensor node number reduction. All of these works promote the development of BSNs in the direction of energy-efficiency and accuracy.

#### 2.1.1. Classification of Sensors

In practical applications, the type of sensors and the number of sensors a BSN system employs depend largely on the particular application scenario and system infrastructure [34]. BSN system may take advantage of many different types of sensors to complete the detection of physiology signals, human behavior, and the surrounding environment. Sensors in BSNs can be of many types due to their various application-specific requirements.

According to the types of measured signals, sensors in BSNs can be divided into two categories [37]. The first category, which includes accelerometers, gyroscopes, ECG sensors, electro-encephalograph (EEG) sensors, electromyography (EMG) sensors, visual sensors, and auditory sensors, collect continuous time-varying signals. This type of sensor collects signals continuously, placing more emphasis on real-time signal acquisition, and correspondingly both data transmission quantity and power consumption are very large. The second category, such as glucose sensors, temperature sensors, humidity sensors, blood pressure monitors, and sensors monitoring blood oxygen saturation, collect discrete time-varying physiology signals. As the signals that sensors collect change slowly, the amount of data transmission quantity is smaller than for the first category. It is possible to reduce energy consumption by using sleeping mode.

According to the types of data transmission media, the most commonly used sensors in BSNs can be divided into the following three categories: wireless sensors, which employ wireless communication technologies such as Bluetooth or Zigbee, radio frequency identification devices (RFID), and Ultra Wideband (UWB) to communicate with other sensors or devices. Most applications employ this type of sensors for improving wearability and reducing the interference of sensors with usual activities. Wired sensors, employing wired communication technologies, can replace wireless sensors if wearability is not seriously affected. The transmission mode is more stable than that of wireless sensors. However, their installation and deployment is relatively complicated. Removing wires completely will be an inevitable trend for BSNs [13]. Human body communication (HBC) sensors, which use the human body as the transmission medium, have only been proposed in recent years. This type of sensor adopts sub-GHz frequencies without antennae, which reduces the power consumption and the size of sensor nodes. Therefore, they can easily be integrated into body-worn devices. What's more, the communication distance of a HBC sensor is constrained around human body, which effectively improves the communication security [38]. However, it has less communication speed than a normal wireless sensor. Recently it was supported by the IEEE 802.15.6 standard for use in short-range, low-power and highly reliable wireless communication systems which are close to, on or in the human body [39].

According to the deployment positions of sensor nodes, sensors in BSNs can be divided into three categories [37]: Type 1 are wearable sensors, such as temperature sensors, pressure sensors and accelerometers. The size and weight of the sensor should be considered in the design process, in order not to interfere with the usual activity of users. Type 2 are implantable sensors, which can be implanted or inhaled/ingested into the body, such as a camera pill. This type of sensor needs to be not only tiny enough, but also non-corrosive and biocompatible. Type 3 is placed surrounding people, and can be used to recognize behaviors and collect information about the surroundings, such as visual sensors.

According to their automatic adjustment ability, sensors in BSNs can be divided into two categories: self-adapting sensors, which can automatically adjust processing method, order, and parameters, boundary conditions or constraints according to data characteristics, make themselves adapt to the statistical distribution and structural characteristics of the measured data, in order to get the best treatment effect. Non-self-adapting sensors, which are simple to design and need no consideration of self-adjusting function, are widely used in BSNs at present. Because of the requirements of complexity and accuracy enhancing, self-adapting methods will be gradually applied to design of sensors.

#### 2.1.2. Main Researched Sensors

With the development of different applications, more and more sensors are appearing in the BSN field. The most commonly used sensors are shown in Table 1. Several typical BSN sensors are introduced below.

(1) ECG Sensors
  ECG sensors are used for electrocardiograph signal monitoring, which is the main way to diagnose heart disease in electronic healthcare systems. ECG signals reflect the change of current intensity on the skin caused by the contractile activity of the heart over time, which can easily be recorded using non-invasive electrodes on the chest or limbs of body [40]. They can be represented as a pattern of cyclically occurring ECG waveforms with different frequency contents: QRS complex, P and T waves. ECG waves indicate the overall rhythm of the heart and weaknesses in different parts of the heart muscles, and they can be measured to diagnose abnormal heart rhythms. Among ECG waveforms, the QRS complex reflects the electrical activity within the heart during ventricular contraction and provides much information about the state of heart [41]. ECG sensor nodes transmit data in a wireless way, which contributes to better wearability. Generally, the nodes are so small that they can easily be embedded in fabric or plaster.
  According to the types of electrodes, ECG sensors can be divided into three categories: sensors with wet electrodes, sensors with dry electrodes, and sensors with non-contact electrodes. Wet electrodes were used for ECG monitoring first, but now are rarely used in BSNs, as this kind of electrode may cause skin irritation and signal degradation due to dehydration. As an alternative, dry electrodes have become more and more popular, but they still contact the skin directly. In addition, dry electrodes which don't have the benefit of conductive gel, are much more sensitive to the conditions of the skin and are highly susceptible to motion artifacts (MAs). In Reference [42], a kind of wireless sensors with non-contact electrodes is presented. The sensor consists of a set of capacitive electrodes manufactured on a standard printed circuit board that can work within fabric or other insulation. In contrast to wet and dry contact sensors, non-contact sensors do not require any direct contact with the body, so they are completely insensitive to skin conditions. Nevertheless, the denoising ability requirement is relatively high for non-contact sensors. Experiments show that insulating layers between non-contact sensors and skin may obscure the features of smaller signals, such as P-waves, if the layers are too thick to maintain signal quality. Moreover, insulating layers may cause noise too, which must be weakened or removed by more effective denoising algorithms.
(2) Accelerometers
  Accelerometers are used for measuring the acceleration of components in an inertial three-dimensional coordinate system. They play an important role in human energy expenditure detection and behavioral recognition, because of their small size, relatively low cost, as well as the convenience of integrating them into existing sensor network platforms [22].
  One purpose of utilizing accelerometers is to detect human energy expenditure. Accelerometers can obtain physical activity frequency, motion intensity, and other information if placed on the human body. Energy expenditure can be inferred by a series of algorithms based on the information accelerometers get. Compared with other methods utilizing passometers or cardiotachometers alone, this method has higher accuracy and has become a trend in energy expenditure detection [43]. At present, the most accurate energy consumption instrument is called the LivePod [44], which employs triaxial accelerometers to identify the action of a human body in any direction by an intelligent fuzzy algorithm. It can achieve an accuracy of 97% using a high-precision personalized model for energy consumption calculation. Compared with the Livepod, the accuracy of existing research models needs to be improved.
  Compared with human energy expenditure detection, behavioral recognition is much more complex, because it employs not only accelerometers, but also other inertial sensors to recognize direction and angle of movement and other action parameters. Furthermore, it needs to get activity information from raw sensor data, thus the signal processing process is more complex [45]. Generally, accelerometers work together with gyroscopes and magnetometers to achieve a more accurate measure, but it's not absolute. In Reference [46], a kind of inertial measurement unit (IMU) called EcoIMU which only utilizes accelerometers is presented, which can accurately measure linear acceleration and angular velocity. It is built from a pair of triaxial accelerometers, expanding the application scope of accelerometers from relative motion tracking to absolute motion tracking. Moreover, the use of accelerometers significantly lowers the power consumption and the cost of an IMU, which conforms to the desirable low-power characteristics of BSNs. Reference [22] indicates that behavioral recognition often requires multiple sensor nodes. The more nodes it utilizes, the higher accuracy it obtains, however the more difficult it becomes. Therefore, in order to identify motions accurately with only a few nodes, finding the best deployment location for sensor nodes has become a hot topic of research in the field of BSN sensors in recent years.
(3) Pressure Sensors
  In BSN applications, pressure sensors are generally used to monitor pressure changes of the underside of foot in real-time mode, providing data for pressure analysis, behavioral recognition, and energy expenditure detection. Because of its special position on the human body, it is difficult to integrate pressure sensors with other modules, such as wireless communication modules, in one sensor node. They are generally installed on a pressure plate or inside special insoles. The pressure sensors are always connected with an external micro-controller by cable. In Reference [47], a low-cost foot pressure measurement system has been developed to measure the pressure of each contact point of the pelma by a pressure plate, in order to calculate the contact area. The system adopts feature extraction and pattern recognition techniques to recognize clubfoot pattern, aiming to automatically design personalized insoles. In Reference [48], researchers place pressure sensors into insoles to calculate motion parameters, including distance, time, total weight, speed, and frequency. The parameters are used to recognize behaviors aided by feature extraction and support vector machine (SVM), providing a novel method to measure energy expenditure. However, the proposed method ignored the problem of temperature drift caused by motion, which affects the accuracy of pressure measurements. This is a considerable problem in pressure sensor design.
(4) Respiration Sensors
  Different from the sensors introduced above, respiration sensors in BSNs are generally composed of several sensors, such as pressure sensor, accelerometer or gyroscope. They get respiration parameters indirectly by detecting the expansion and contraction of the chest or abdomen based on those sensors. Respiration sensors are always used in the treatment of respiratory diseases and for continuously monitoring human symptoms. A breathing feedback system with wearable textile-based sensors is introduced in Reference [49]. The sensor gets the depth and respiratory rate by recognizing expansion and contraction of the chest based on a kind of piezoresistive material called a carbon-loaded elastomer. It can be implanted into fibers or textiles, helping users to practice correct breathing and to treat respiratory diseases. In addition, Reference [50] proposed a new method to solve the problem of continuous respiration monitoring. This method is based on reconstruction of the angular motion caused by breathing, which makes use of accelerometer data. It tracks the axis of rotation and obtains the angular velocity of the sensor nodes placed on the chest, implementing accurate respiration detection despite the interference caused by body movements. However, most respiration sensors monitor the parameters of breath simply, with rare consideration of flow rate and tidal volume of respiration, which is the future research direction of respiration sensors.

#### 2.1.3. Design of Sensor Nodes

Although sensors are the key components of sensor nodes, they cannot work independently. They must work together with other modules to realize signal acquisition. In order to meet the demands of low power consumption and high wearability, many issues need to be considered in the sensor node design process, including energy control, fault diagnosis, and reduction of sensor nodes.

(1) Energy Control
  In order to implement long term monitoring functions, energy control has been one of the hot topics in the field of BSN sensors for a long time. Presently, primary researches include low-power architecture design, low-power processor design, low-power transceiver design, and energy acquisition design. In Reference [19], a programmable architecture of ultra-low power consumption based on dynamic time warping designed specifically for wearable inertial sensors is proposed. Provided that the sampling frequency is 3 Hz and bit resolution is 4 bits, the sensor node can run at a power consumption of 9 μW, meeting the need of recognizing motions perfectly. The power consumption of a sensor node can be reduced more than three orders of magnitude by using the proposed architecture compared to traditional low power microcontrollers, such as the MSP430. In Reference [21], researchers introduce a low-power processor design, which reduces supply voltage to below threshold voltage, in order to lower leakage power and prolong the life of logic circuits and SRAM. Consequently, it is a compelling strategy for energy-constrained systems with relaxed performance requirements. However, the effects of process variation become more prominent at low voltages, particularly in deeply scaled technologies. For this purpose, the paper presents a system-on-a-chip which demonstrates techniques to mitigate variation, utilizing timing methodology to avoid output voltage failures and propagation delays in logic gates, in order that the system can run at a low voltage. In terms of low-power transceiver design, Reference [51] presented a low power contact impedance sensor node named CIS, integrating an efficient transceiver using human body as a kind of transmission medium. The proposed CIS adopts a capacitive sensing technique based on LC resonance for detecting the parasitic capacitance between electrodes and the human body to turn a transceiver on or off automatically. It can compensate for channel quality degradation due to contact impedance variation, thus leading to significant reduction of the power consumption of a Low Noise Amplifier (LNA) by more than 70%. In addition, energy acquisition design can help the sensor nodes to collect power by themselves. For example, biological fuel cells can convert chemical energy into electric energy using a biocatalyst. In Reference [15], researchers mention that glucose biological fuel cells can collect power by enzymatic chemical reactions occurring in the human body.
(2) Fault Diagnosis
  BSNs consist of multiple sensors, while any broken down sensor may affect the running of the whole system, so failure node detection and isolation cannot be ignored. One solution is to detect them by comparing the contents among neighbor nodes. Reference [20] indicates that detection accuracy decreases as the number of sensor nodes decreases. Furthermore, the number of nodes in a BSN is relatively small, and there is rarely a circumstance applying multiple sensors of the same kind to collect signals at the same position, so the method mentioned above is more suitable for WSNs rather than BSNs, and it does not perform well in the latter. Another challenge is to find the right neighbors for data validation in order to reduce false alarms. In Reference [52], authors propose a more reasonable approach, which uses sliding window techniques to split a sensor stream into segments and provides a fault detection algorithm based on these segments. The proposed fault detection algorithm can be divided into two sub-categories: history based and non-history based. The fault detection rate of the former is more reliable, while the latter can help to validate the former.
(3) Reduction of Sensor Nodes
  Reduction of sensor nodes mainly aims at inertial sensors for behavioral recognition. It not only improves the wearability of behavioral recognition systems, but also lowers the cost, saves energy, simplifies the recognition process, as well as reduces data redundancy. Principal methods to solve the problem are the optimization of node placement and the improvement of activity recognition algorithms. At present, some research projects regard node placement optimization as one of the main research problems in the field of behavioral recognition in BSNs. In Reference [22], the authors propose several schemes for how to place wearable sensors for different body actions and provide an optimal framework of node placement. The framework can also help to find the most relevant band from all the time-frequency features collected by wearable accelerometers. In this paper, the authors divide the analytical method process into three steps: feature extraction, feature selection, and classification. Then they classify the actions by different data features, and provide the best placement scheme for each type of action. Different from the method mentioned above, Reference [53] proposes a method to reduce the number of sensor nodes by improving the activity recognition algorithms. The authors have also proved that the problem is NP-hard. However, they propose a behavioral recognition model which uses a decision tree structure to minimize the number of nodes involved in classification of each action. The method reduces nodes by 72.4%, while still maintaining 93.3% classification accuracy.

### 2.2. Trends and Challenges

With the deepening of the research, sensor technology is becoming mature with its application scope expanding on a daily basis. It's developing towards the direction of high wearability and low power consumption. As a BSN is characterized by long-term real-time monitoring, measures must be taken to reduce the size of sensors and eliminate any possible physical and chemical harm to the human body for the sake of long-time use. Many studies have been carried out on the improvement of wearability in previous research. However, despite the fact a small portion of sensors show good wearability, many others still need to be improved. Especially for sensors monitoring human activities, there are still a lot of work to do in order not to affect everyday life and sports training. Similarly, solving the problem of power consumption of sensors is still a long-term goal. Nowadays, the functions of sensor nodes are becoming more and more complicated, and the demands of data processing quality increase continuously as well, which makes prolonging the service life of sensor nodes another major problem for the future, and meanwhile low-power design methods are being studied. In addition, with new physical parameters of interest being discovered continuously, researchers should design more and more new types of sensors. Meantime, research into new materials and new energy in other fields will promote the progress of BSN sensors.

There are still some difficulties in the research of BSN sensors, such as extension of sensor life, improvement of measurement accuracy and design of implantable sensor antennas. In practical applications, replacement of sensor node cells may not be timely, and sometimes impossible, especially for implantable sensor nodes. How to supply energy is a great challenge to achieve long-term automatic monitoring. Some researchers propose acquiring energy from the external environment, but this technology is still not mature. Besides, there exists another challenge on how to improve the accuracy of signal measurement, when accuracy depends on the position of sensors and the denoising ability of sensors, so finding new denoising methods is also a challenge at present. As for implantable sensors, it is hard to design the antenna of a wireless communication module, as the antenna material must be non-corrosive and biologically compatible, such as platinum and titanium, but the wireless communication capability of these materials is less than that of copper, while their cost is too high to be widely used [54]. Meanwhile, the radio frequency (RF) environment will change with the variation of wearer's age, weight, and posture. All of these factors above will affect the quality of wireless communication, which needs to be considered carefully by designers [55].

## 3. Data Fusion

Data fusion in BSNs is a procedure for processing data or information coming from multiple sensors with multi-level, multifaceted processing to make data more effective and meet users' needs better. BSNs produce a large amount of physiological data according to the application purpose. The way in which these data are manipulated is a fundamental issue faced by sensor node designers. Data fusion techniques combine data from multiple sensors and related information, which can achieve more accurate inferences compared to a single, independent sensor. It also can filter noise effectively, making predictions and inferences from monitored actions or phenomena [56]. At the same time, data fusion techniques can reduce data redundancy, and consequently reduce the load and energy consumption of BSNs, with the advantage of prolonging the network lifetime [57]. Not all data in BSNs needs to be fused, such as blood pressure, body temperature, and heart rate. Data of these types are measured by single sensors, and can reflect physiological performance directly. Other types of data which are measured from multiple sensor in BSNs, like heart waves, pulse waves, and data to identify people's motion, cannot be used directly. In these cases, data fusion techniques are necessary.

There has been a lot of research in data fusion. Data fusion techniques were applied in the military field in early days, including automatic target recognition in the design of smart weapons, guidance of autonomous vehicles, remote sensors, and battlefield supervision. Later, they were applied in nonmilitary fields, such as robotics, automatic control of industrial manufacturing systems, and the development of smart buildings [58]. As a branch of WSNs, BSNs are also developing very fast in the fields of medicine [59], or activity recognition [60], in which data fusion techniques play an important role.

### 3.1. State-of-the-Art Research on Data Fusion

The process of data fusion mainly includes preprocessing, feature extraction, data fusion computation, and data compression. There are always some kinds of noise and interference in human physiological signals, which cause that collected signals cannot be used directly. In this circumstance, preprocessing is helpful, as it can filter noise from data and get useful information. The main preprocessing methods are piecewise linear representation, Fourier Transform (FT), Wavelet Transform (WT), high pass and low pass filters, mathematical morphology filters, Laplacian Transform, and Kalman filter, *etc.*, which can be applied to remove noise. Feature extraction is carried out after preprocessing, which assembles representative data into feature vectors to distinguish among different actions or phenomena [61]. In this stage, researchers analyze information by means of Fast Fourier Transform, wavelet analysis, *etc.*, to thus extract the features of collected signals. After that, the extracted features are used as input of data fusion computation methods which will generate fusion results. Data fusion computation on information of different types is difficult. Researchers found that activity classification algorithms and intelligent computing methods [24,25], including hierarchical methods, decision trees, Bayesian Network (BN), Artificial Neural Network (ANN), and Hidden Markov Model (HMM), are suitable for solving this kind of problem. Furthermore, the methods can effectively relieve the pressure of low recognition accuracy. Finally, data that gets preliminary treatment will be compressed before transmission, which will significantly reduce the total amount of data transmission in BSNs. In this way, power consumption is reduced [62]. In the aspect of data compression, several encoding methods are common techniques. In recent years, research on data fusion has achieved many fruitful results, and a number of research hotspots in the data fusion process area have come out, as well as combination of data fusion with other techniques and applications.

#### 3.1.1. Preprocessing

When detecting human body physiological parameters, data collected by sensors is often affected by interferences, leading to a lot of noise. Reference [40] states that these disturbances are from two sources. Firstly, low Signal to Noise Ratio (SNR) signals caused by irregular movements and human respiratory actions. Secondly, interference with signal acquisition and transmission caused by instrumentation amplifiers in the surrounding environment or in sensors of the system itself. Noise types also vary due to the different types of sensors and different applications. MAs, such as baseline drift, flicker noise, and thermal noise, as well as wire interference may be caused by instrumentation amplifiers in measuring ECG, EEG, and EMG. High and low frequency noise will be introduced during the signal collection process by inertial sensors measuring body movements.

Preprocessing is an efficient and effective way to carry out the denoising process on original data while maintaining useful information. The main techniques include piecewise linear representation, FT, WT, high pass and low pass filters, mathematical morphology filters, and Kalman filter. For example, Discrete Fourier Transform (DFT) and Discrete Wavelet Transform (DWT) are naturally suited for non-continuous data processing [23]. Techniques such as low-pass median value filter, Laplacian Transform, and Gaussian filter can be used to remove high frequency noise. The Kalman filter is an optimal recursive data processing algorithm, which is always used to correct measured values based on the observed value of the current moment and the best estimated value of a previous moment. It plays an important role in dealing with measured values which are not very accurate.

Using dry electrodes is one of the significant factors which increases power line interference (PLI) and MA, and finding solutions to reduce these noises or interferences is an important issue. A lot of methods are proposed to remove the PLI in bio-potential signals, such as the notch filter and analog notch filter [63]. However, the removal of MA in wearable healthcare systems is more complex. Due to the nature of wearable devices, the captured ECG signals are severely distorted with baseline drift and MAs [64]. In Reference [65], researchers introduced the multiscale mathematical morphology filtering concept into QRS detection, while the method of mathematical morphology, initiated in the late 1960s, has become one of the favorite signal analysis tools in many shape-oriented problems [66,67]. They successfully designed the ECG QRS complex detection algorithm based on it. Other researchers use the algorithm in different ways. They utilize hybrid mathematical morphology filtering to suppress impulsive noise when doing opening-closing operations to get a more effective QRS detection [68]. This can remove baseline drift and use modulus accumulation to enhance signals. In Reference [63] a single-scale mathematical morphological (MM) filter and an approximated envelope are combined together. This MM filter removes baseline wand erring, impulsive noise, and the offset of DC componentd while the approximated envelope enhances QRS complexes. These algorithms have been further improved in removing MAs and other interference. In order to realize activity recognition and get accurate characteristic values which are not affected by system noises, adopting a Kalman filter can obtain precise parameters of moving objects, such as instantaneous angular and coordinate data. As data collected from sensors are not absolutely correct when monitoring movement and posture, researchers put the Kalman filter into use for processing data, so as to make output result close to the real data. In a practical application, a Kalman filter is always adopted to process acceleration data [69]. It can also be used in applications such as gait tracking to calculate thurl angle and swing angular velocity.

#### 3.1.2. Feature Extraction

In general, features can be defined as the abstraction of raw data. The aim of feature extraction is to get the feature vectors in a tidy form, representing the characteristics of the original data accurately. Most activity recognition methods use windowing techniques to divide sensor signals into smaller time segments, and features will be extracted from training data [24]. Those features often characterize windows of body-fixed sensor data, and are used as inputs of classifiers.

Commonly used features are as follows [25]: (1) Time-Domain Features: such as Variance and Root Mean Square (RMS); (2) Frequency-Domain Features: such as Spectral Energy, Spectral Entropy; (3) Time-Frequency Domain Features: such as Wavelet Coefficients; (4) Heuristic Features: such as Signal Magnitude Area (SMA), Signal Vector Magnitude, and Inter-axis Correlation; (5) Domain-Specific Features: such as features used in Time-Domain Gait Detection. In ECG detection, the QRS waveform analysis features must be extracted due to the importance of QRS [68].

Techniques commonly used in feature extraction include SVM-Based Feature Selection, K-Means Clustering, and Forward Backward Sequential Search. Besides, Feature Transform Methods include Principal Component Analysis (PCA), Independent Component Analysis (ICA), and Local Discriminant Analysis (LDA). In practical applications, researchers explore many methods to extract more accurate features in addition to the techniques introduced above. Among these feature extraction methods, wavelet analysis is particularly useful in recognizing human activities, for it can identify activity transition points and generate time-frequency characteristics while enhancing the signal. Reference [70] proposes a feature extraction algorithm based on time series approximation which converts the sensor-based time series data into a density map. It realizes fast, accurate and efficient classification of activity, while it can easily be deployed in a BSN at the same time. In Reference [71], the authors propose a systematic approach of feature detection and extraction for measuring human postures based on sensing and feedback. They demonstrate that the construction of sensing and feedback system for dynamic postures is much more complicated than that for static postures. In particular, they consider that dynamic postures can be described by a set of blueprints or human body movement trajectories.

#### 3.1.3. Data Fusion Computing

Data fusion algorithms are the core of data fusion computing. A lot of research has been done in integration of data of the same type. In data fusion algorithm design, modeling information of different types and fusing them together are problems faced by researchers. Techniques or algorithms of fusion computations can be divided into two categories: the activity classification algorithm and the influence model. The former includes threshold-based classification, hierarchical methods, decision trees, and k-nearest neighbor (K-NN), while the latter includes SVM, BN, ANN, HMM. Besides, Gaussian mixture models, Fuzzy Logic, and Markov chains are also in use. Actually, two or more techniques are usually applied in BSN applications.

Activity analysis is an important field of data fusion computation in BSNs. It records activity patterns in a period of time which relies on a classification algorithm to recognize different types of activities by analyzing data coming from wearable sensors [24]. Reference [72] adopts a supervised learning approach to recognize 14 physical activities, using a binary decision-tree with a Naive Bayes classifier at each internal node. It also develops a feature selection algorithm based on mutual information to process data from 14 tri-axial accelerometers, which can recognize human activity with a 96% average accuracy. Threshold-based classification could differentiate static postures and dynamic activity, and identify dynamic activities of different types, such as discriminating between going up and down stairs from walking straight [73]. Nowadays, the methods of threshold-based classification and robustness algorithm are being combined together and applied to the detection of falls. The authors of Reference [45] use three kinds of classifier, which are AdaBoost, HMM, and K-NN, to analyze data from accelerometers when identifying human hand activity. The result shows that AdaBoost can achieve the highest recognition accuracy (86%). The drawback of these classifiers is that they need a large amount of training data to ensure accuracy of recognition.

In recent years, researchers have done a lot of research on influence models, and the major research directions include ANN, BN and HMM. ANN is used as a solid clustering algorithm, and it works like the biological nervous system of a brain that consists of a large number of small and simple interconnected components, that is, neurons. Each neuron has a certain amount of computing power and a strong learning ability, which enables it to easily add new context information into the system. The WSN system based on ANN will inevitably produce noise, but it can still work well, which shows the good nature of ANN. Moreover, the unsupervised training on input data can be realized effectively [74]. Self-Organizing Map (SOM) is a kind of ANN method. In addition to the advantages inherited from ANN, SOM provides an efficient way of visualizing and clustering data. However, SOM has the features of instability and dependence on the training data. To solve these problems Reference [28] proposes a novel Spatio-Temporal Self-Organizing Map (STSOM). Compared with traditional SOM, STSOM not only minimizes the number of neurons, but also reduces the iterations of the learning and classification processes, which enable the system to run locally in a low-power way.

BN is one of the most effective theoretical models for uncertain knowledge representation and reasoning. A BN is a directed acyclic graph. Nodes represent a random variable, while directed edges between nodes represent the relationship between nodes. BNs use conditional probability to represent the relationship between the various information elements and can effectively conduct expression and integration of multi-source information. In the study of BN, it follows an independence assumption that each node in graph is strictly independent from other nodes. However, in BSN applications, the assumption is challenged by the increase of dependency between parent and child. In Reference [56] it is proposed that if hidden nodes are inserted into BSNs, system stability can be enhanced, while Reference [75] has reported some similar work about introducing hidden nodes by using a distributed Bayesian network. References [24] and [56] indicate that hidden nodes can be used to coordinate the inherent redundancy of sensor networks and are able to detect node failures. Furthermore, irrelevant sensor data is not sent across the network, which will reduce power consumption. In recent years, researchers have proposed a multi-sensor data fusion algorithm based on D-S evidence theory, taking D-S theory as an alternative of Bayesian inference, which is a methodology for the representation and combination of empirical evidence. In Reference [76], researchers design a BSN system for human posture recognition based on the algorithm. Experimental results show that the system can achieve recognition accuracies between 98.5% and 100%.

As a kind of Markov chain, HMM is a probabilistic model used to represent non-deterministic processes, which consists of states, actions, and observations. Generally, in applications of activity recognition, it is required not only to sense and simulate the context of static scene, but also to identify the dynamic scene, therefore the HMM is applied to the supervising layer, generating a model of context transitions, in order that the analysis and identification of dynamic activities in real world can be realized. HMM plays an important role in the study of dynamical influence models of human interactions. In Reference [77], researchers collect the social behavioral signals of each entity in a social network using various sensors, modeling by the signals and time stamps based on HMM. They then infer the underlying pattern of interpersonal influence. Furthermore, the paper points out that HMM can also speculate on the functional roles of people and make classification accurately. In Reference [78], researchers develop Graph-Coupled Hidden Markov Models (GCHMM) for studying the model of local infectious disease spread in a social network, analyzing the transition dependencies between multiple HMM. This paper discusses a case of infectious disease which spreads among students, collecting and processing information by mobile phones. The model can forecast the spread path of an infectious disease, so as to control its spread.

#### 3.1.4. Data Compression

After preliminarily treatment of signals, sensor nodes generally do compression and encryption rather than sending it to a base station or sink node directly. Studies show that data compression can effectively reduce the amount of data transmission. Since the memory module and data sending module consume lots of energy [26], data compression can lower power consumption effectively on BSN sensors, by reducing information stored in memory and transmitted by the transceiver.

In BSNs, data compression can be achieved by not only classical data compression algorithms, such as source encoding, differential encoding, and Huffman encoding, but also some novel methods proposed by several researchers. Some collections and transmissions of physiological signals have instantaneity requirements, so minimizing the amount of data transmission is necessary. For example, Reference [62] proposes a compression algorithm based on interception and differential encoding techniques, because the temporal and spatial of data collected by inertial sensors have correlations. Experiments prove that the algorithm can effectively reduce the amount of data transmission and give better real-time performance. Reference [79] adopts a algorithm called joint orthogonal matching pursuit (JOMP) based on distributed compressive sensing (DCS) theory, combining it with the synchronous mechanism, which can control interval times and efficiently reduce the number of samples, the amount of data processing, transmission and storage. In Reference [80], researchers evaluate the effectiveness of a wireless neural recording system using compressed sensing for transmission. They propose a union of support techniques for compressive sensing. Compared to conventional basis pursuit reconstruction, the proposed technique provides an average signal-to-noise and distortion ratio (SNDR) improvement of 6 dB and a maximum SNDR improvement of 9.5 dB. It also provides a 2-fold reduction of the output data rate. Reference [81] adopts the DFT method, and solves compression and decompression problems by discrete sinc interpolation. In the ECG monitoring application, Reference [82] proposes a compression algorithm based on WT, which achieves low transmission delay by reducing the size of frames. Similarly, in Reference [83], researchers also study the data compression of ECG, and propose a low-cost quadratic compression algorithm which can increase encoding speed and reduce energy consumption effectively, while maintaining high signal quality. For the sake of minimizing the energy consumption of a multi-hop wireless network based on joint routing and compressed aggregation, Liu Xiang *et al.* [84] analyzed the complexity of this optimization problem and find that it is NP-complete. They further propose a mixed-integer programming formulation (MIP) to obtain the optimal aggregation trees for small scale problems, along with a greedy heuristic algorithm to obtain the near-optimal aggregation trees for large scale problems. Finally, the energy efficiency is improved.

### 3.2. Trends and Challenges

In the field of data fusion research, there exist some research trends, including data processing position, lightweight data fusion algorithms, design of pervasive context-aware frameworks, as well as cloud computing. As communication consumes more power than local computation, wireless data transmission uses a large amount of power [26]. Therefore, processing data locally is an efficient way to save power. However, the data processing ability of nodes is generally limited, which leads to a contradiction about data processing localization. How to solve this problem is a hotspot for further research. At the same time, limited resources and computation ability make it difficult for nodes to carry out large scale computing, and therefore designing lightweight embedded data fusion algorithms to alleviate the load of processors is necessary. In addition, BSNs usually operate in many different situations, while the human body has diverse signs and actions, and so does the environment. Studies on adaptive and distributed BSN frameworks to adapt to these cases is necessary. In Reference [85], researchers designed a distributed framework based on a Markov decision process for coordinating sensing among sensors in a BSN system. The framework can generate a globally optimal sampling policy by analyzing the status of each sensor. It solves the problems that occur when the sampling rates of sensors are too high or too low, so as to extend the overall lifetime of the BSN system. With the development of the cloud computing technique, mining data streams of body sensors online or offline with cloud technology has become a new research hotspot. In Reference [86], researchers integrate cloud computing into BSNs, providing flexibility of data storage solutions using cloud technology. They globally realize data analysis, storage and access for BSN services based on cloud computing.

There are still some difficulties in data fusion of BSNs. Affected by human activities and equipment, signals of sensors are inevitably mixed with some noise, which leads to the fact that data collected by sensors cannot be used directly [27,87]. How to effectively filter all kinds of noise is now a big challenge. In practical BSN applications, there is a certain degree of redundancy among data collected by multiple nodes. Collaborative data fusion could effectively reduce the amount of data and improve calculation accuracy. To figure out the relevance of heterogeneous data is difficult, therefore, designing a better collaborative data fusion algorithm becomes another challenge. Furthermore, the combination of BSN techniques and cloud computing increases the difficulty of data management and calculation. Large scale computing frameworks design, real-time data processing and data stream management have all become major challenges.

## 4. Network Communication

BSNs have many features similar to WSNs in the aspects of network communication, such as in the aspects of network architecture, radio technology, communication protocols, energy control, and network security, *etc*. As a result, many of the BSN technologies are derived from WSN technologies which are more mature [31,88–90], but some characteristics of BSNs make it difficult to directly transplant technologies from WSNs. For example, sensor devices are basically deployed on or in a human body, so the electrical characteristics of the body can influence radio communications. Human activities will also cause frequent changes of network topology [89,91]. Therefore, researchers should improve the existing WSN technology to apply in BSN communications properly, or propose new technology to meet the specific needs of BSN communications.

### 4.1. State-of-the-Art Research on Network Communication

In the process of BSN designing, many factors should be taken into consideration. Consequently, such designs as network topology, physical layer, MAC layer, and routing layer can be better accomplished. In order to improve the efficiency of network protocol, in some cases, boundaries among layers need to be broken, and cross-layer protocols need to be introduced.

#### 4.1.1. Design Factors

In BSN design, a lot of research work has been carried out, and the following aspects should be seriously considered. Generally, BSN nodes cannot employ circumscribed wired power, which is a constraint for long time operation. Meanwhile, nodes are designed as small as possible for wearability, so their battery capacity is very limited. For example, the sensor node designed by Benny *et al.*, is only in 26 mm size [92]. Some other researchers adopt the energy acquisition technology harvesting power from surrounding electromagnetic environment instead of batteries, but this leads to a stricter power consumption restrictions at the same time [16]. In BSNs, many key applications cannot work well if any node stops working when the energy has run out. Therefore, the network architectures and protocols should guarantee that nodes could complete the data transmission of highest quality with the least power consumption. In addition, BSNs should balance power consumption distribution, avoiding the overload of a few nodes.

Radio transmission range of sensor nodes will become smaller along with miniaturization design and energy conservation design. Examples of ultra-low range transceivers in the literature include Reference [93] with 0.2–1 m, Reference [94] with 0.2 m and Reference [95] with 0–1 m transmission ranges. The smaller the transmission range is, the more careful the network topology design should be. In this circumstance, the star topology is not suitable, because nodes far away from sink node are not easy to communicate with.

The path loss directly reflects the network communication performance. In BSNs, the electromagnetic waves transmit by diffraction along the body instead of penetrating into the body, which causes a remarkable path loss, especially when transceivers are placed on the different sides of body, *i.e.*, one on the chest and the other on the back [91].

Many BSN applications such as fall detection have strong reliability demands [96]. When a fall happens, the whole system must have the ability to collect the fall information immediately, and then send alarms to the appropriate consoles, but if the network encounters heavy congestion or topological partition at the same time, data could not be send out, which will affect the system on making a decision for reaction. In this case, users may be hurt. Therefore, the number of errors of data acquisition must be as small as possible, and so must the delay.

Due to the fact the data collected has a close relationship with individual information, it will inevitably involve user data security issues. If personal information leaks, the user will be in danger. Cryptography and trust evaluation methods can effectively solve these issues. In Reference [97], researchers propose a key agreement scheme called ECG-Improved Jules Sudan (ECG-IJS), which is suitable for BSNs. It allows neighboring nodes to share a common key generated by the features which are extracted from ECG signals. The proposed scheme can improve the security of data communication and avoid previous key distribution. In addition, because of the fact that BSNs are susceptible to a variety of node misbehaviors, traditional trust evaluation methods are vulnerable to attacks. Recognizing malicious behaviors and excluding malicious nodes need to be considered. Reference [98] presents a distributed trust evaluation model with the feature of application-independent, which allows each node to evaluate integrated trust values of others based on continuously monitoring the behaviors of its neighbors. Trust management is executed by cryptographic techniques such as symmetric encryption/decryption and hash operations. The proposed model can effectively detect and exclude malicious nodes. Similar to the previous study, an attack-resistant and lightweight trust management scheme called ReTrust is proposed in Reference [99]. It adopts a two-tier network architecture in which network nodes are divided into two parts: sensor nodes and master nodes. The former is responsible for data collection, while the latter is responsible for computation and communication. In this architecture, a master node is used to compute the trust values of all sensor nodes within its range, which can identify malicious behaviors effectively, so as to avoid collaborative attacks. Besides security issues, there are many issues related to human health. For example, some sensor devices are deployed on or in human body, so radio radiation, sensor working temperature, cutaneous stimulation from electrodes and some other factors may injury the human body [16,100].

Since human body is moving constantly, sensor nodes on or in the body have the feature of mobility. Along with body movements, the distance between sensor nodes will constantly change. This will cause serious topological partition problems in that some links between nodes are broken and built frequently. This kind of network is called Delay Tolerant Network (DTN), whose routing protocol is very difficult to design and realize [89].

#### 4.1.2. Network Topology

The network topology affects the system performance features, such as power consumption, traffic load, node robustness, selection of MAC protocol, and routing protocol. The common network topologies include star topology, mesh topology, ring topology, and bus topology. Both ring topology and bus topology are not fit for deploying on a complex dynamic human body. According to the literature statistics, there is almost no application systems using these two topologies because the BSN scale is small and sink nodes need to gather body information to send it to remote computers. Hence, the star topology has been very popular since BSNs came into being [101–103]. All the nodes directly communicate with the sink node. The network communication protocol realization is relatively simple, and sometimes even a routing protocol is unnecessary for data transmission, so the star topology is widely used. In practical applications, Personal Digital Assistants (PDAs) are often used as sink nodes which communicate with other nodes, completing data processing, data transferring, and part of decision-making [104]. The star topology has some disadvantages which limit its further development in BSNs, for it is difficult for the nodes far away from the sink node to communicate with it due to energy limitations. Besides, the nodes located in different sides of body will fail in building connections because of low data reception rates [91]. Different from the star topology, the mesh topology with more complex realization but more superior performance, is more suitable for multi-hop networks. It can reduce path losses caused by diffraction. Each node just needs a small amount of energy to communicate with its neighbors, and power consumption of whole network is well-distributed. Currently many researchers are focused on multi-hop networks, in which couples of new technologies are well applied, such as energy acquisition, human body channel, *etc.*, so as to provide better BSN services. The differences between star topology and mesh topology are summarized in Table 2 [29].

#### 4.1.3. Physical Layer

The physical layer, as the bottom layer of BSN structure, is mainly responsible for encoding and decoding of signals, preamble generation and removal for synchronization, bit transmission and reception, and specification of the transmission medium. In the past few years, the research on BSN physical layer has focused on two aspects, which are channel selection and channel characterization.

**Figure 3.**
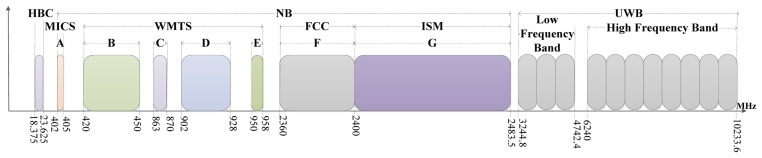
BSN frequency bands.

(1) Channel Selection
  The physical channel, as the data transmission medium, plays a key role in the BSN. Its characteristics directly affect the performance of the whole network. Therefore, in order to achieve the best transmission performance, the most suitable channel must be chosen according to different data transmission scenes. Figure 3 presents all the bands in a BSN. The Human Body Communication (HBC) band which takes the human body as the data transmission medium is only used by BSNs. Its primary technology is electric field coupling which includes capacitive coupling and galvanic coupling [105,106]. An HBC channel of the capacitive coupling type is developed based on the near electric field around body which is induced by a transmitter terminal, and a receiver terminal is used to detect the weak coupling changes of the near electric field along the human body channel. Research has shown that the data rate of capacitive coupling is up to 2 Mb/s [107]. In addition, Reference [108] has put forward a kind of electromagnetic coupling technology which takes human body as the electromagnetic waveguide to finish communications. Medical Implant Communications Service (MICS), which has a transmission power of 25 μW, is also one of communication bands suitable for low data rate networks [109]. The Wireless Medical Telemetry Service (WMTS) is mainly used in wireless telemetry in hospitals. Its band is similar to some parts of the local TV spectrum in the United States, but no research on interference between the two bands has ever been done so far. Band F is the 2.36 GHz medical band newly approved by the Federal Communication Commission (FCC) in the United States. The Industrial Scientific Medical (ISM) band commonly used in BSNs operates at 2.4 GHz. Hence, for the sake of avoiding interference, corresponding solutions which could deal with interference must be designed [110]. UWB communication is believed to have great advantages and is promising in WBAN applications. It is a technique with low-power and high data rate features. Its large bandwidth signals provide robustness to jamming with low probability of interception. Moreover, UWB can be used to monitor vital respiration and heart-rate parameters [111–113]. In addition, UWB has good penetrating properties that could be applied to imaging in medical applications [114].
  In 2007, the Sixth Working Group of IEEE 802.15 was established to standardize on BSN bands. The team eventually divided the bands into three parts: UWB, NB, and HBC [115]. MICS, WMTS, FCC, and ISM belong to NB. In addition, with the newly proposed IEEE802.11ad standard, researchers try to present novel techniques to enable over-body propagation between 15 GHz and 40 GHz, and even the unlicensed 60 GHz region [116].
(2) Channel Characterization
  Accurate channel characterization can improve the quality of applications, such as estimating delay and reducing path loss. In recent years, another key BSN physical layer research area is the channel characteristics of various bands. The IEEE802.15.6 working group has put forward the fundamental content about channels and their characterization. There are three common channel characterization methods:
  (a) Measurement-based methods. Fabio *et al.*, have measured the narrow band transmission channel, and analyzed the channel characteristics including the average path spreading gain, large-scale attenuation, and small-scale attenuation. Results have shown that movement, location, and environmental factors can cause path losses. Movements of the human body will result in shadows [30].
  (b) Simulation-based methods. Reference [117] makes 3D scene simulation of signal propagation in the channel by means of Finite-Difference Time-Domain (FDTD). It employs a leap frog algorithm to alternately calculate the electric field and magnetic field in the space-domain and simulate the electromagnetic field changes by time-domain updating. This simulation model is of great applied value, according to the comparison between simulation results and measured data.
  (c) Combination with simulation and actual measurement method. Reference [118] has provided a path loss model which can be used to evaluate the energy performance in single-hop and multi-hop networks. The sensor nodes deployed around human body can measure the sent and received data among different parts of human body, then import the collected data into a 3D model of human body, and numerically study path losses. Finally, the path loss parameters and time-domain channel characteristics can be obtained.

#### 4.1.4. MAC Layer

The original purpose of the BSN MAC layer was to achieve maximum throughput, minimum delay, and to maximize network lifetime by controlling the main sources of energy waste, *i.e.*, collisions, idle listening, overhearing, and packet cost. Generally, MAC protocols are grouped into contention protocols, schedule protocols, and contention-schedule protocols [31]. In contention MAC protocols, such as Carrier Sense Multiple Access/Collision Avoidance (CSMA/CA) protocols, nodes contend for the channels to transmit data. If the channel is busy, nodes will defer their transmission until the channel becomes idle. These protocols are not demanding on strict time synchronization, but they cause extra cost. Sana *et al.* studied the behavior of CSMA/CA protocol in BSNs and concluded that CSMA/CA protocol encounter serious collision problems for high traffic nodes [119]. In schedule protocols such as Time Division Multiple Access (TDMA) protocols, the channel is divided into several time slots. These slots are assigned to nodes and each node transmits during its corresponding slot. These protocols are energy conserving protocols. There is no contention, idle listening, and overhearing problems, but these protocols require frequent time synchronization which is somewhat difficult to achieve. An enhanced Distributed Queuing Medium Access Protocol (DQ MAC) based on TDMA has been proposed in Reference [102]. It proposes a remarkable improvement of overall network energy efficiency, which scales well for very dense BSNs and is particularly suitable in medical scenarios. H-MAC is also a TDMA-based MAC protocol designed for BSNs, which aims to improve energy efficiency by exploiting heartbeat rhythm information to perform time synchronization, unlike other general MAC protocols which achieve synchronization by sending beacons [104]. WhMAC, a signal- oriented TDMA-based protocol of low power, utilizes WBSN-specific features and a novel ultra-low power wake-up receiver, to achieve flexible wireless data transfer of physiological signals [120]. BodyMAC, another TDMA-based MAC protocol, with a flexible bandwidth allocation strategy, improves node energy efficiency by reducing radio transmission, idle listening, and control packets overhead times, to realize the smallest collision possibility [121]. Contention-schedule MAC protocols combine the characteristics of the former two mentioned above. The authors of [122] designed a traffic load aware sensor MAC for collaborative BSNs, called ATLAS. The protocol takes the superframe structure of 802.15.4, while it makes full use of its Contention Access Period (CAP), Contention Free Period (CFP), and Inactive Period (IP). The protocol can provide better energy efficiency, higher capacity-utilization, and less delay based on adaptive traffic load evaluation.

In addition, part of the information of a BSN is correlated. For instance, a patient suffering from a fever triggers temperature, blood pressure, and respiration sensors at the same time [101]. The instant data increase may result in serious network congestion. In this case, a CSMA/CA protocol encounters serious collisions and extra energy consumption. Besides, nodes are required to perform Clear Channel Assessment (CCA) before transmission, which will make the situation worse, but TDMA-based protocols can provide good solutions to the issues mentioned above [31].

#### 4.1.5. Routing Layer

Although MAC protocols can solve many problems in BSNs, they do not cover addressing and end-to-end package delivery problems which rely on routing protocols. In BSNs, the development of routing protocols needs to consider the following issues: firstly, the available power of each node is very limited, and power consumption is not balanced among nodes. Along with the continuous power consumption when running, part of the nodes consume more power while others consume less, which will result in an unbalanced remaining power distribution and reduce the life of the entire network. Some researchers focus on resolving this issue of unbalanced energy distribution. Reference [32] presents a heuristic self-adaptive routing algorithm for rerouting connections among nodes in an energy constrained on-body network. It can select the reachable parent node automatically to balance energy distribution. This protocol can help disconnected nodes reconnect again and minimize network transmission delays.

Secondly, the topological partition problem is another issue that BSN routing protocols must take into consideration. The body movements will cause frequent partitioning or disconnection in BSN topologies. In addition, this situation gets aggravated by the ultra-short wireless range due to its wearable design [123]. To solve the problem of topological partition, Quwaider *et al.*, have put forward a probabilistic packet routing mechanism in Reference [89]. LLF, a stochastic metric called link likelihood fact, is calculated in each node using the history of the link quality between nodes in the network. This metric which could reflect the postural trend of the human body, can be used for decision-making in forwarding the packets to neighbors. In fact, the routing goal is to reduce end-to-end delays by choosing the highest likelihood links. Another method to solve the topological partition problem is to employ routing protocols based on replication which make several copies of a packet to send to several proper nodes and thus increase the chances of arrival. These protocols will definitely cause more serious network congestion and delay, due to excessive packet transmissions. For ultra-resource-constrained BSN, such overheads are not acceptable. Majid *et al.*, have proposed a store-and-forward data routing method by adopting a gossiping strategy to improve the performance of replication-based protocol [124]. Different from the random method, a predefined part of the data is selected to be sent, which offer a better solution to the frequent topological partition problem in large scale BSNs.

Finally, WSNs almost never consider temperature factors. However, the effect of sensor temperature in BSNs cannot be ignored, since the implantable sensors usually transmit biomedical data to neighboring nodes from time to time which may cause damage to the human body due to high sensor temperatures. At present, a lot of researchers study algorithms by means of thermal-aware routing to deal with temperature factors. Reference [125] proposes a Thermal-Aware Routing Algorithm (TARA) protocol defining hotspots as areas where nodes have a relatively high temperature. In order not to cause a temperature rise of these areas any longer, TARA attempts to establish another route to detour around the hotspots using a withdrawal strategy. Unlike TARA, the Least Temperature Routing (LTR) protocol always chooses neighboring node which has the lowest temperature as its next stop [126]. This protocol algorithm is very simple, since LTR does not initially schedule the route of packets and just choose nodes with the lowest temperature instead and the packets will basically detour to the destinations. In fact, LTR is a greedy approach, which may be locally optimal, and probably cannot become globally optimal. One variant of LTR protocol is the Adaptive Least Temperature Routing protocol (ALTR) [126]. Different from LTR, ALTR keeps the number of hops of each packet. Once it exceeds a predefined maximum hop count, ALTR will apply the shortest hop-routing protocol as an alternative protocol to forward packets to the destination as soon as possible. Reference [100] proposes the Least Total-Route-Temperature (LTRT) protocol. It calculates routes and transmits packets by the single-source shortest path algorithms, such as the Dijkstra algorithm. However, the weight in graph theory is denoted by temperature of nodes in order to detour the nodes of high temperature. A detailed comparison of BSN routing protocols is shown in Table 3.

#### 4.1.6. Cross Layer

In WSNs, cross layer design is an effective way to improve system performance by compressing or adopting the redundant information across layers and modules. Cross-layer optimization combines cognitive science, artificial intelligence, and convex optimization to allocate resources, so as to efficiently improve the utilization of these resources in wireless networks. Meanwhile, it can also offer better QoS for nodes in wireless networks [127]. Reference [128] proposes a cross-layer Dynamic Source Routing (DSR) algorithm which extends DSR by exploiting cross-layer optimization techniques to minimize the frequency of recomputed routes. DSR initiates a route discovery only when a link failure occurs, thus leading to a 50% reduction of recomputed routes compared with the traditional DSR protocol, and finally improves the routing energy efficiency of WSNs. Such a study also exists in BSN research. Reference [129] studies the real-time data streaming applications in BSNs. Firstly, researchers formulate routing and residual energy of nodes during the establishment of the routing paths. And then a cross-layer optimization is applied for improving the real-time performance of sensorial feedback for users, maximizing the lifetime of system at the same time. Furthermore, they also study the ARBA protocol, which monitors the complex conditions in data streaming from various sensors in a real-time mode, adaptively allocating resources to cope with changes in context and application requirements. Reference [130] presents a cross-layer communication protocol, called Cascading Information retrieval by Controlling Access with Distributed slot Assignment (CICADA), designed for wireless multi-hop mobile BSNs. It uses the same packets to take care of both medium access as well as routing, which makes low delays and energy efficiency possible while preserving network flexibility. BSN-MAC [101], another cross layer protocol, can make dynamic adjustments based on the feedback provided by sensors to achieve a better energy efficiency performance by the interaction of network coordinators and sensors. Reference [33] presents MOFBAN, a lightweight Modular Framework for Body Area Networks, which is a special cross layer design. The framework supports many features, such as MAC control, routing and robustness, realized by modules, unlike a normal layered architecture, so it is adaptable and expandable.

### 4.2. Trends and Challenges

There are still many issues that need to be further studied in BSN communication. Minimizing power consumption as well as ensuring quality of communication links is a long-term objective, and energy-efficient MAC and routing protocold remain to be developed [54]. IEEE 802.15.6 has defined many BSN band standards, so developing many upper layer protocols which support a variety of physical layer channel is an inevitable trend [35]. Besides, in some medical applications, the key information should be sent out in time when a medical emergency occurs, but BSN data correlations often cause network congestion which makes network protocol design that supports emergency communication become a major challenge in further BSN development. Finally, the deployment of BSNd is different from that of traditional sensor network applications. They should be able to deal with topology changes and adapt to conversion between in-body and on-body. Besides, nodes should be ready to join or withdraw from network, so node context awareness and network configuration is also a trend in future studies [34].

There are still many challenges in the BSN network communication research field. First of all, experimental channel models which are significantly simplified fail to take full account of human motion and environmental changes. At the same time, as personal safety issues it involved, it is really difficult to apply implantable or wearable devices to applications. There are still many factors which can affect channel characteristics, such as the electrical characteristics of the human body, human mobility, and energy restrictions. Accurate descriptiond of channel characteristics and channel prediction are very difficult too. Secondly, for MAC layer protocols in BSNs, TDMA is obviously more suitable than CSMA/CA [31], but the time synchronization mechanism is still a weakness of TDMA. Thirdly, because BSNd have the features of small scale and low-power transmission, a simple human body action will cause a relatively big change of topology with old connections being disconnected or new connections built, which is a challenge for the routing protocol designer. Moreover, although cryptography technology can achieve better security performance, due to limited sensor resources and the fact that pre-deployed secret keys are hard to replace, many existing key management and distribution algorithms are not suitable for BSNs. Therefore, designing efficient key management, distribution and agreement schemes in BSNs is still challenging [97]. Finally, the trade-off between energy efficiency and the complexity of fast routing algorithms also needs to be figured out [35]. All of above are big challenges for BSN topology design.

## 5. Applications of BSNs

**Figure 4.**
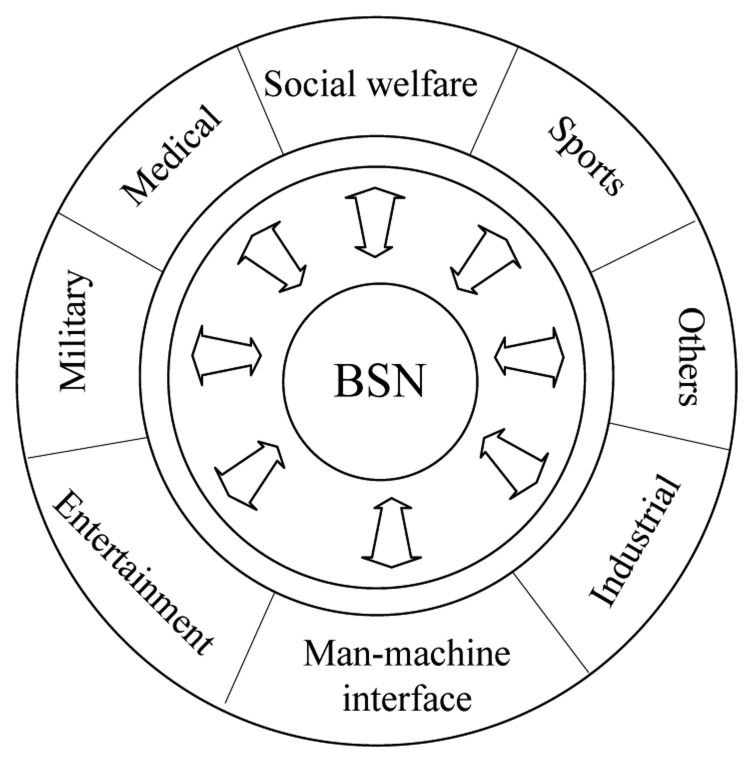
Application fields of BSNs.

With the deepening and extension of research, BSN technology is gradually becoming mature and widely used in many fields, including medicine, social welfare, sports, and man-machine interfaces, as shown in Figure 4. In addition, in other fields, such as the military, entertainment, and industrial systems, applications of BSNs can also be found.

### 5.1. State-of-the-Art Research on BSN Applications

#### 5.1.1. Medical Field

BSN is mostly used in medical field and has a widespread applicability for many kinds of diseases. Traditional clinical monitoring is generally carried out in hospitals, where patients' condition may be affected by the clinical environment and monitoring frequency. In contrast, monitoring based on BSNs can be carried out in a family environment, which makes the results closer to reality.

Cardiac disease diagnosis by ECG signals monitoring is a common application of BSNs. In Reference [1], researchers present a wearable ECG acquisition system. The system adopts the Planar-Fashionable Circuit Board (P-FCB) technique, and screen-prints the electrodes directly on fabric, which enables long-term monitoring without skin irritation. The electrodes have high conductivity and adhesiveness, and can be attached on the skin surface. Another kind of monitoring method is to integrate sensors into an adhesive plaster. The plaster can be attached on the skin, which has the advantages of convenience and low cost. For example, Reference [131] suggests integrating all the sensors on one plaster, achieving a smart poultice with a reconfigurable sensor array. A thin flexible battery is integrated on the plaster, which improves wearability. However, this kind of power supply mode leads to great reduction of the plaster's lifetime. Reference [132] proposes a method utilizing wireless power technology on sensor nodes, which can remove batteries and solve the lifetime problem, and further improve the wearability of the monitoring system. In addition to cardiac disease diagnosis, Parkinson's disease (PD) monitoring is also one of the main applications of the BSN technique in the medical field. Because the behavioral recognition techniques of BSN can provide long-term monitoring and credible data, it is more persuasive than the monitoring by clinical observation. Meanwhile, the monitoring can be carried out continuously during daily life, so the patients' information is collected in real time. It is helpful to judge the severity of disease and provide scientific support for therapy. Behavioral recognition techniques have been widely used in many of the existing PD monitoring systems. For example, in Reference [133], researchers identify movement characteristics associated with Parkinson's patients by wearable sensors, and achieve real-time monitoring with high accuracy. In recent years, many studies have shown the connection between PD and speech impairment. Some researchers have proposed a wide range of speech signal processing algorithms (dysphonia measures), which has become a new trend for predicting the severity of PD symptoms. In Reference [134], researchers investigated the accuracy of speech signal processing algorithms which are used to discriminate Parkinson's suffers. Result shows that the classification accuracy can reach almost 99% based on only ten dysphonia features. Respiratory disease treatment can also be implemented with the help of BSN technology. It utilizes a respiratory sensor for monitoring depth and frequency of breathing, so as to guide patients to take correct breathing training, which plays a very important role in respiratory disease rehabilitation [49,50]. In addition, [3] studies the condition of patients recovering from surgery, employing an ear-worn sensor for post-operative monitoring of patients both in terms of their respiratory function (oxygen saturation and heart rate), as well as their mobility (accelerometer data), to judge whether patients have abnormal symptoms. This application reduces the risk of complications of operation patients during the rehabilitation period. In Reference [135], researchers study lung disease by integrating sensors on a shirt. They measure trans-thoracic bioimpedance outside the body by an electrical impedance tomography technique, thereby measuring lung liquid volume. The application achieves the prediction of pulmonary edema and prevents lung disease from becoming worse.

Nowadays, mobile communication devices such as mobile phones and tablet computers are playing more and more important roles in people's daily lives. Some studies have introduced mobile devices into the medical field and realized real-time monitoring of patients with the help of the embedded sensors, data processing and wireless communication modules of mobile devices. This kind of technique is named mobile health (m-health) which is a new direction of BSNs in the medical field in recent years. Because of the adoption of ready-made mobile devices, m-health techniques do not need to develop additional dedicated devices, which effectively reduces system costs and increases wearing convenience. In some m-health applications, mobile phones are deployed as data aggregators or intermediate servers for lightweight processing. In Reference [136], researchers collect physiological signals from ECG sensors, accelerometers and temperature sensors by an Android smartphone, which works as an aggregator. The signals are shown on the screen of smartphone in the form of either graphical or text notations. At the same time, data streams are transmitted to a central server with Bluetooth for storage and analysis. Reference [137] presents a preventive healthcare system called myHealthAssistant, which also uses a mobile phone as an aggregator. The presented system can capture heart rates and calorie expenditures by accelerometers and heart rate sensors, helping to control daily activities as well as specific gym exercises. Moreover, the system can record people's daily activities into a SQLite database and generate a fitness diary. In Reference [138], researchers present a system called Intelligent Mobile Health Monitoring System (IMHMS), which can provide medical feedback through mobile devices based on biomedical and environmental data. The system collects temperature, blood pressure, glucose and other signals with a variety of sensors. All the signals are transmitted to a mobile device which is in charge of sending data streams to a medical server. After that, a patient's health status is predicted intelligently based on data analysis and then transmitted back to the patients by a medical server. Finally patients can take necessary actions depending on the feedback. In some other researches, mobile phones can be used as a platform which concentrates sensors and processor. In other words, researchers capture signals by sensors in the mobile phone and carry out signal processing with the processor of the mobile phone. Reference [139] presents a method for accurate activity recognition that only uses one phone with an accelerometer in it. Different from other activity recognition methods, the phone-based method removes additional accelerometers deployed on different locations and avoids analyzing data by a personal computer, which is convenient for users. Previous phone-based methods assumed that the acceleration signals are collected from a known fixed location and orientation. However, varying the location and orientation can affect the recognition effect in a real world situation. For dealing with the issue, a project-based method for device coordinate system estimation is proposed. Researchers make a detailed comparison of experiments with different device locations, and determine the best location of a mobile phone, which can achieve a recognition accuracy rate of about 90%.

#### 5.1.2. Social Welfare

Along with the ceaseless improvement of social welfare, highly intelligent electronic systems are needed to help long-term monitoring of patients, the disabled, the elderly, and children. Some BSN applications utilize behavioral recognition and context awareness technology to better the quality of care for vulnerable groups.

In a study of electronic monitoring system for daily activities, Reference [140] proposes a behavioral recognition method which mainly focuses on the classification results of human body posture conversion, achieving daily activity monitoring of patients and the elderly. As fall-detection is also very important, in Reference [141], researchers utilize wearable inertial sensors to predict fall actions. A threshold detection method is introduced based on the magnitude of inertial frame vertical velocity as the main variable to separate fall activities from non-fall ones. The algorithm is able to detect all fall events at least 70 ms before impact, thus proving the feasibility of the application. As a more in-depth research, Reference [142] develops a wearable airbag incorporating a fall-detection system that uses both acceleration and angular velocity signals to trigger inflation of airbag, which prevents serious injury caused by falls. In Reference [143], its authors study fall-detection from another perspective. They design a kind of sensor called ear-worn activity recognition (e-AR) with the ability of predicting the risk of falls by gait and balanced detection, which is more wearable than others mentioned before. In addition to taking care of people with disordered movements, electronic monitoring systems are widely used in nursing care of people with cognitive impairments. In Reference [9], researchers design an action reminder system for dementia patients, which utilizes a sensor node arrangement in the environment and on the body to recognize patients' actions and trigger different levels of alarm. Similarly, Reference [144] designs a kind of adaptive recognition system. The system can recognize behaviors as well as prompt patients with visual and auditory messages. In Reference [10], researchers achieve real-time detection of dirty diapers by context awareness technology. It can remind nursing staff to replace diapers and solve the problem of nursing inconvenience caused by urinary incontinence, and effectively improve the quality of nursing.

#### 5.1.3. Sports

In the field of sports, BSN is mainly used for motion recognition and physiological status detection, which can help athletes with scientific training, posture correcting and skill improvement. A multimodal remote sensing platform called TennisSense is introduced in Reference [4]. The platform applies wireless inertia monitor units placed on arm, aided by network digital cameras and a set of UBISENSE 3D tracking sensors placed around the tennis court. The sensors can capture and recognize athletes' actions, and then evaluate and correct them. For the same purpose, Reference [5] proposed a system for kinematic analysis of swimming strokes. The system utilizes an inertial sensor mounted on the swimmer's goggles for acceleration measurement, which can calculate parameters such as the pitch and roll angles from recorded acceleration. Reference [6] develops a framework for the use of inertial sensors as a low-cost putting coach for golf. The framework offers great help to golf players on the promotion of movement precision when swinging clubs. In the field of sports, in addition to behavioral recognition, other BSN techniques can also be used to improve the training quality. For example, in Reference [145], researchers design a sweat pH monitoring system which can measure the pH of sweat in real time by placing barcodes made of novel ionic liquid polymer on the surface of body, which achieves non-invasive detection of the physiological state at the time of moving. Reference [146] presents an alternative approach for heart rate measurement which uses sound signals received from a microphone instead of an ECG sensor method. It can estimate the athlete's physical status from the heart rate, and help athletes train scientifically and avoid injuries caused by electrodes in direct contact with the skin.

#### 5.1.4. Man-Machine Interfaces

Along with the development of behavioral recognition technology, BSN devices can be used to replace traditional man-machine interfaces, such as keyboards, mice, joysticks, and touch screens, and they can also be used to design somatosensory control systems. In addition, BSN technology can assist handicapped people in daily life. In Reference [147], researchers propose some intra-body communication (IBC) applications. For instance, an IBC-enabled sensor embedded inside the shoes of a blind person can send voice information to the user, telling him or her the current location, identifying doorways or crosswalks. IBC-enabled eye-glasses can help deaf people comprehend audio broadcast announcements, by displaying texts converted from sound with the help of IBC-enabled speakers, Reference [148] presents a hand-gesture-based glove for facilitating communication among people with speech and hearing-impaired disabilities. In the system, a wireless sensor glove equipped with five flex sensors and a 3D accelerometer is used as input device. By integrating the speech synthesizer into an automatic gesture recognition system, user's hand gestures can be translated into sounds. In addition, Reference [149] proposes a method using visual implant technology to recover visual perception of the blind or patients with retinal degeneration, which is also an application of BSN in the man-machine interface field.

### 5.2. Trends and Challenges

The scope of BSN applications is becoming wider and wider, which reflects not only in the broadening of the application domain, but also the improvement of service quality. Precisely, the improvements include three aspects: wearability, power consumption, and generality. Moreover, in the aspect of implementation, except for adopting normal sensors, bio-sensors, base stations and other hardware devices, more and more researchers are getting interested in mobile devices with embedded sensors such as mobile phones and smartphones.

Wearability is one of the most important issues of BSNs. Researchers have done a lot of work on improving features like the volume, weight, wear resistance, softness, and biocompatibility of sensor nodes. In the process of sensor node design, reduction of volume and weight can be achieved by optimizing system hardware architecture and applying micro-electronic components. In order to enhance the wear resistance of sensors, highly abrasion-resistant materials are generally adopted to protect them. In the aspect of improving softness, P-FCB technique was proposed and is still under continuous study. Besides, flexible batteries can also improve softness, on condition that the problem of the great reduction of energy storage is solved. The improvement in biocompatibility could contribute to avoiding inflammation and allergy, as well as reducing the failure rate of devices. References [150] and [151] show how the defects of biofouling, hermeticity of encapsulation, electrode passivation and limited life of the immobilized enzymes can cause implanted sensor failure and affect patient health. Therefore, designing sensors with mild side effects is an important research topic to improve wearability.

The power consumption problem, which is one of the major research directions now and in future, exists in any BSN system. Different from WSN ones, BSN sensors are small in size. They can only use compact batteries with limited capacity, which lead to short sensor node lifetimes [14]. At the same time, in many BSN applications, especially for those using implantable sensors, it is hard to prolong usage time by replacing the batteries. In view of these problems, how to reduce power consumption of sensor nodes, and increase the capacity of a battery in a limited volume, as well as prolong system life-span by energy acquisition from the surroundings are all potential development directions. In fact, the problems of power consumption are more than limited battery capacity and energy acquisition. In the aspects of circuit design, data processing algorithms and network communication protocols, a lot of work has been done and needs to be done sequentially and in depth in the future.

In the future applications of BSNs, it is required that not only a system can work in any environment but also that data sharing and function cooperation can be achieved among different systems, therefore many studies focus on how to improve the universality of BSN applications. Universality mainly consists of algorithm universality and system universality. As for improving algorithm universality, further study is needed to explore the issues of signal processing applied to multiple non-standard data sets and finding the basic algorithms suited for many conditions. In order to improve system universality, research indicates that shaping an eventual data standard for data sharing can promote function cooperation among BSN applications.

As mobile phones are becoming more and more popular in daily life, m-health technology has attracted extensive attention in medical field over the recent years. Existing research has implemented activity recognition, energy consumption monitoring and other functions. More and more functions will be gradually discovered in the future. In addition, the method of adopting mobile devices in BSN systems is not only suitable for the medical field, but also for other fields. With the development of smartphones, the capabilities of processors are becoming increasingly powerful, and so are smartphone operating systems. With the help of mobile phones, more complex, diverse and humanized functions will be achieved, which is a significant trend of BSNs in the short run.

In recent years, BSN theory has undergone significant development, but there are still many challenges in practical applications. For a lot of BSN products it is difficult to meet design requirements. In the conversion process from technology to product, defects of usability and accuracy limit BSN technology used in some important fields such as the military and serious medical field.

The usability of BSNs consists of many aspects and needs to be considered appropriately. Robustness is the base of usability. BSN systems should be able to diagnose faults and achieve failback by themselves [2]. If the network topology of a BSN changes, it should be able to correctly handle problems of node connectivity. Users' privacy issue is also very important. The application must ensure that privacy data can only be retrieved by authorized users. The data should be encrypted during transmission and storage [25]. In addition, much interdisciplinary knowledge is needed in the studies of wearability, lifetime, and service intelligence *etc.*, which is difficult.

The accuracy of a BSN system depends on the process of signal acquisition, denoising, feature extraction, fusion calculation, data compression, transmission, and other follow-up analysis. However, it is difficult to make sure that all the processes are accurate. Although current BSN applications can achieve some of the basic functions, the accuracy is still not good enough to fully meet application requirements. For example, the fall-prediction system proposed in Reference [141] may mistake some special movements for falls and pop the air bag unexpectedly, which will seriously affect usual activities. However, if overemphasizing accuracy while designing a system, it would be inevitable to achieve other target such as low computational cost and low power consumption [152]. Large amounts of work need to be done to balance the relationship between computational cost and accuracy of a system. The introduction of simpler mathematical models to reduce the computational cost and power consumption in the future is an effective way to accomplish all this.

## 6. Conclusions

BSNs, arranging sensors in, on and around the human body, realizes the detection of human action and physiological information, which has been widely used in the fields of health care, social welfare, sports, entertainment, *etc*. The ubiquitous network is coming with the method of taking human body as a part of the communication network. Therefore, BSN has broad application prospects and market potential.

Although BSNs have been a hotspot of research with the emergence of many practical applications, many open problems still remain. For example, in the design of sensor nodes, more attention should be paid to node size minimization and energy consumption reduction. Designers should place emphasis especially on avoiding the danger of damage to human tissues caused by the heat generated by sensors implanted in the human body. When designing data processing algorithms and communication protocols, the same problem of power consumption should be solved, as well as the network robustness problem resulting from moving nodes. In addition, due to the close relationship between BSNs and human health, there are topics of moral and legal constraints in actual applications. In short, BSNs represent the integration of multi-disciplinary areas, aiming to solve many key issues in several of these areas, which requires researchers to do further exploration.

This paper describes the concepts, origin, architecture, and research areas of BSNs, with a comprehensive introduction to the current applications and issues of BSNs. We focus especially on the research status, development trends and challenges of sensor technology, data fusion technology, and network communication technology. Besides, there are some more aspects to be further improved and summarized, such as data security and service quality.

## Figures and Tables

**Table 1.** Commonly used sensors in BSNs.

**Sensors**	**Function**	**Signal Type**	**Sampling Frequency**	**Placement**
Accelerometer	Obtaining acceleration on each spatial axis of three-dimensional space.	Continuous	High	Wearable
Artificial cochlea	Converting voice signal into electric pulse and sending it to implanted electrodes in ears, generating auditory sensation by stimulating acoustic nerves.	Continuous	High	Implantable
Artificial retina	Receiving pictures captured by external camera and converting them to electric pulse signals, which are used to stimulate optic nerves to generate visual sensations.	Continuous	High	Implantable
Blood-pressure sensor	Measuring the peak pressure of systolic and the minimum pressure of diastolic.	Discrete	Low	Wearable
Camera pill	Detecting gastrointestinal tract by wireless endoscope technique.	Continuous	High	Implantable
Carbon dioxide sensor	Measuring the content of carbon dioxide from mixed gas by infrared technique.	Discrete	Low/Very low	Wearable
ECG/EEG/EMG sensor	Measuring voltage difference between two electrodes which are placed on surface of body.	Continuous	High	Wearable
Gyroscope	Measuring angular velocity of rotating object according to principle of angular momentum conservation.	Continuous	High	Wearable
Humidity sensor	Measuring humidity according to the changes of resistivity and capacitance caused by humidity changes.	Discrete	Very low	Wearable
Blood oxygen saturation sensor	Measuring blood oxygen saturation by absorption ratio of red and infrared light passing through a thin part of body.	Discrete	Low	Wearable
Pressure sensor	Measuring pressure value according to the piezoelectric effect of dielectric medium.	Continuous	High	Wearable/Surrounding
Respiration sensor	Obtaining respiration parameters indirectly by detecting the expansion and contraction of chest or abdomen.	Continuous	High	Wearable
Temperature sensor	Measuring temperature according to the changes of materials physical properties.	Discrete	Very low	Wearable
Visual sensor	Capturing features of subject, including length, count, location, and area.	Continuous/ Discrete	High/Low	Wearable/ Surrounding

**Table 2.** A comparison between star topology and mesh topology.

	**Star Topology**	**Mesh Topology**
Path Loss	Nodes on the same side with low path loss. Nodes on the different sides with high path loss.	Reducing path loss caused by diffraction though multiple hops.
Radio Transmission Range	Not suitable for small radio propagation range.	Adjusting radio propagation range by changing the number of nodes
Energy Consumption	Nodes closer to sink node consume lower power.	The nodes nearer to sink node consume more energy, as they have to forward not only their data but also data from other nodes.
Transmission Delay	Sensors connect with sink node directly take the least possible delay in transmission.	Nodes closest to sink node get their data quickly, without any intermediate delay.
Inter-User Interference	Nodes farther away from sink node need higher power to transmit data with more interference to other nodes.	As each node only transmits to its neighbors, the energy of transmission is low and hence with smaller interference.
Node Failure and Mobility	Only the failed node is affected and the rest nodes of network perform well.	The whole network including nodes with errors need to be reset.

**Table 3.** Summary of existing BSN routing protocols.

**Protocol**	**Content**		**Resolved Issues**
FPSS	Choosing path intelligently among nodes based on heuristic self-adaptive algorithm in energy constrained on-body network.		Energy balance
PRPLC	Forwarding packets to proper neighbors by prediction of postural trend based on link likelihood fact.		Topological partition
TARA	Establishing route to detour around hotspots area using a withdrawal strategy.		Minimizing the thermal effects of Implanted biosensor
LTR		Always choosing neighboring node with the lowest temperature as next stop.	
ALTR	Choosing next stop by both the lowest temperature node and the shortest hop count.		Implanted biosensor
LTRT	Choosing the shortest path based on a Dijkstra algorithm with the weight of temperature.		Implanted biosensor
